# The impact of organisational factors on treatment outcomes for those seeking alcohol or other drug treatment: A systematic review

**DOI:** 10.1111/dar.13653

**Published:** 2023-04-02

**Authors:** Breanne Hobden, Megan Freund, Samuel Lawson, Jamie Bryant, Justin Walsh, Lucy Leigh, Rob Sanson‐Fisher

**Affiliations:** ^1^ Health Behaviour Research Collaborative School of Medicine and Public Health, College of Health, Medicine and Wellbeing, University of Newcastle Newcastle Australia; ^2^ Equity in Health and Wellbeing Program, Hunter Medical Research Institute Newcastle Australia; ^3^ Clinical Research Design and Statistics Hunter Medical Research Institute Newcastle Australia

**Keywords:** alcohol‐related disorders, substance abuse treatment centres, substance‐related disorders, systematic review

## Abstract

**Introduction:**

Organisational factors have been found to be associated with health outcomes in a number of health‐care settings. Despite likely being an important influence on the quality of care provided within alcohol and other drug (AOD) treatment centres, the impact of organisational factors on AOD treatment outcomes have not been extensively explored. This systematic literature review examines the characteristics, methodological quality and findings of published studies exploring the association between organisational factors and client AOD treatment outcomes.

**Methods:**

Medline, Embase, PsycINFO and the Cochrane database were searched from 2010 to March 2022 for relevant papers. Studies meeting the inclusion criteria underwent quality assessment using the Joanna Brigg's Institute critical appraisal tool for cross‐sectional studies, followed by data extraction of key variables pertaining to the aims. A narrative summary was used to synthesise the data.

**Results:**

Nine studies met the inclusion criteria. Organisational factors examined included cultural competency, organisational readiness for change, directorial leadership, continuity of care practices, service access, service to needs ratios, dual diagnosis training, therapeutic optimism and the funding model/health‐care system that treatment was delivered in. Outcome measures included duration, completion or continuation of treatment; AOD use; and patient perceptions of treatment outcomes. Seven out of nine papers found a significant interaction between at least one organisational variable and AOD treatment outcomes.

**Discussion and Conclusions:**

Organisational factors are likely to impact treatment outcomes for patients seeking treatment for AOD. Further examination of the organisational factors that influence AOD outcomes is needed to inform systemic improvements to AOD treatment.

**Key Points**

- Organisational factors were associated with improved outcomes in seven out of nine studies.
- Included studies had wide variation in alcohol and other drug treatment outcome measures.
- Unit size, care continuity and funding were significantly associated with outcomes.
- Organisational factors are a significant area for future alcohol and other drug treatment research.

## 1 INTRODUCTION

Alcohol and other drug (AOD) treatment clinics provide a range of services from counselling to detoxification across outpatient, residential and community care settings [1]. Between 2019 and 2020, it is estimated that more than 21 million people received AOD treatment in the United States [2], 139,000 in Australia [3] and over 270,000 in the United Kingdom [4]. While treatment outcomes for AOD use are generally positive, they remain modest and variable [5, 6, 7, 8, 9]. Less than 60% of the clients receiving AOD treatment will become abstinent or improve their functioning following treatment [10, 11], and relapse rates in the year following treatment completion are high [9, 12, 13]. Variability in client treatment outcomes not only affects the health and wellbeing of the client, but also impacts the cost‐effectiveness of treatment delivery.

Attempts to understand the variability in effectiveness of treatment for AOD use has led to research exploring both the client and treatment‐level factors associated with successful AOD treatment. Both pharmaceutical and psychosocial treatment level factors have also been extensively researched, with the effectiveness of psychosocial treatments ranging from low‐moderate to high‐moderate [14, 15, 16] and pharmaceutical treatment being modest [16, 17]. McKay and Weis conducted a systematic review examining client predictors of successful AOD treatment and found level of substance use before treatment, as well as severity of psychiatric illness were significant predictors for outcomes [18]. Another systematic review, examining client‐level predictors of successful treatment for alcohol use, also  found levels of baseline dependence to be one of the most consistent individual‐level predictors of successful treatment outcomes for alcohol dependence, as well as addiction severity; psychopathology ratings; alcohol‐related self‐efficacy; motivation; and treatment goals [19]. A recent systematic review by Sliedrecht et al. supported previous findings, demonstrating that significant predictors of alcohol use disorder relapse included psychiatric comorbidity, condition severity, alcohol craving, other substance use, as well as physical health and social factors [20].

In other areas of health care, organisational factors have been suggested to significantly impact clients' treatment outcomes. Organisational factors refer to the operational attributes, processes or conditions within an organisation. Organisational level factors include structural variables (e.g., location or funding model) and process variables (e.g., patient to staff ratios or staff education and training) [21]. For instance, better treatment outcomes (i.e., lower adverse outcomes and mortality) have been demonstrated for hospitals located in urban locations with higher case‐loads [22, 23, 24]; higher staff to patient ratios [25, 26, 27]; specialised treatment units [28]; and teaching status [29]. Despite likely being an important influence on the quality and consistency of care, the organisational level factors that impact AOD treatment outcomes have not been extensively explored [30]. One recent systematic review explored professional and organisational factors in relationship to AOD treatment outcomes [31]. This review, however, limited the organisational factors to five specific aspects of the clinician and organisational workforce, including: years of clinical experience; level of education/qualifications; staff turnover; staff‐to‐client ratio; and professional development. Further the 12 included studies were primarily focussed on adolescent AOD treatment or were conducted more than 15 years ago. While reviews exploring specific factors allow targeted information to be derived, other organisational factors may also have important impacts on client outcomes in AOD treatment settings. A more expansive search, inclusive of a wider range of organisational factors is needed to identify additional important organisational factors that have been explored within the AOD field. A comprehensive examination of organisational factors associated with outcomes for clients seeking treatment for AOD use has not been previously conducted.

### 1.1 Aim

This systematic literature review examines the characteristics, methodological quality and findings of published studies exploring the association between organisational factors and client AOD treatment outcomes.

## 2 METHOD

### 2.1 Data sources

Medline, EMBASE, PsycINFO and the Cochrane databases were searched for relevant studies from 2010 to the date of the search (10 March 2022). A year restriction of 2010 onwards was applied to the search strategy to maximise the likelihood of relevance of findings for current organisational systems and practice. Searches were restricted to studies published in English.

### 2.2 Search strategy

The search strategy was developed in collaboration with a research librarian to best approximate the context domains included in the Theoretical Domains Framework [32]. The Theoretical Domains Framework enables the identification of influences on health professional behaviour, particularly in relation to the implementation of evidence‐based recommendations [32]. AOD use search terms were mapped to headings specific to each database to ensure the relevant literature was captured. Examples of the search terms used were: ‘alcoholism’, ‘substance‐related disorders’, ‘organization and administration’, ‘delivery of health care’, ‘leadership’, ‘attitude of health personnel’, ‘Professional‐patient relations’, ‘education’, ‘health service administration’, ‘patient‐centered care’, ‘financial management’, ‘diffusion of innovation’, ‘cultural competence’, ‘guideline adherence’, ‘physician incentive plans’, ‘treatment outcome’ and ‘outcome and process assessment’. Data S1, Supporting Information, includes the detailed search strategy.

### 2.3 Inclusion criteria

Publications were included if they: (i) examined organisational factors contributing to AOD treatment outcomes (see Table 1 for a priori list of included and excluded organisational factors); (ii) were descriptive studies (i.e., to ensure no intervention impacted the organisational associations on patient outcomes); (iii) reported patient data relating to AOD treatment outcomes (e.g., abstinence, consumption); and (iv) examined AOD treatment clinics.

**TABLE 1:** Included and excluded organisational factors^a^

Included factors	Excluded factors
Professional knowledge Professional skills Professional role and identity Professional beliefs about capabilities Professional attitudes (optimism) Professional beliefs about consequences Professional reinforcement Professional intentions Professional goals Staff ratios (cognitive overload/emotion) Environmental context and resources Workplace pressures (social influences) Professional mental health/morale Cultural competency Private vs public funding	Government level initiatives (e.g., Medicaid change) Differing treatment programs (e.g., comorbidity treatment, parenting education) Screening, brief intervention and referral to treatment Differing treatment modalities (e.g., web or phone‐based) Therapeutic relationship

^a^ Derived from the Theoretical Domains Framework, an examination of the literature and discussion with the behavioural science team.

### 2.4 Exclusion criteria

Publications were excluded if they: (i) exclusively examined patient or treatment factors contributing to treatment outcomes; (ii) were conducted in general practice, emergency departments or general hospital settings; (iii) were intervention, qualitative, non‐databased or case studies; or (iv) specifically examined adolescent treatment seekers (i.e., >50% were aged <18 years). While case studies are considered descriptive research, these were excluded as they are not able to rigorously measure associations between independent and dependent variables. Adolescents were excluded as the needs and profiles of adolescent treatment seekers are likely to differ to the needs of adults and therefore the organisational factors relating to treatment outcomes may also differ [33].

### 2.5 Screening of articles

An initial review of titles and abstracts was performed by two authors (Breanne Hobden and Samuel Lawson) to identify potentially relevant studies. The first 50 papers returned from the search were assessed by both authors to determine agreement for inclusion (88% agreement rate; κ = 0.4505; *p* < 0.001). Discrepancies were discussed between authors. Titles and abstracts for the remaining articles were then screened for inclusion by one author (Samuel Lawson). Two authors (Breanne Hobden and Samuel Lawson) reviewed the full text of the same 10 potentially included publications and assessed their agreement rate (90%; κ = 0.6154; *p* < 0.0175). Discrepancies were discussed until agreement was reached. Following this, the authors independently reviewed the full text of remaining papers to determine inclusion. Article screening was performed and reported in line with the Preferred Reporting Items for Systematic Reviews and Meta‐Analyses guidelines [34].

### 2.6 Quality assessment

Quality assessment was conducted using the Joanna Briggs Institute critical appraisal tool for cross‐sectional studies [35]. The tool examines study quality across eight domains (see Table 2). Each study was independently assessed by two authors (Breanne Hobden and Jamie Bryant or Megan Freund and Justin Walsh), with disagreements resolved through discussion. A statistician (Lucy Leigh) checked the components of the critical appraisal tool relating to quality of the sample size, statistical analysis and response rate for each paper.

**TABLE 2:** Joanna Briggs Institute critical appraisal of methodological quality for cross‐sectional studies.

First author, year	Criteria for inclusion in the sample clearly defined	Study subjects and setting described in detail	Exposure measured in valid and reliable way	Objective, standard criteria for condition measurement	Confounding factors identified	Strategies to deal with confounding factors stated	Outcomes measured in a valid and reliable way^a^	Appropriate statistical analysis
Guerrero, 2011	■	■	●	‡	■	■	●	■
Guerrero, 2016	■	■	■	■	■	■	■	■
Guerrero, 2017	■	■	‡	■	■	■	■	■
Myers, 2022	■	■	N/A	■	■	■	●	■
Ritter, 2021	■	■	■	■	■	■	■	■
Schaefer, 2011	■	■	■	■	■	■	■/●	■
Schulte, 2010	‡	■	‡	■	■	■	■	■
Shin, 2011	■	■	■	●	■	■	●	■
Witbrodt, 2012	■	■	■	■	■	■	‡	■

Abbreviations: ●, no; ‡, unclear; ■, yes; N/A, not applicable.

^a^ Two or more icons indicates multiple outcomes.

### 2.7 Data extraction

Data extraction was undertaken by two authors (Breanne Hobden and Samuel Lawson) and included: author; year; country; setting; eligibility (for centres and patients); sample (total number of participants, age and gender); treatment seeking substance; organisational factors (including how measured); outcome measures and follow‐up time point(s); and outcomes.

### 2.8 Data synthesis

A narrative summary approach was undertaken due to the low number of included studies and the heterogeneity of organisational factors and study outcomes.

## 3 RESULTS

### 3.1 Search results

**FIGURE 1:**
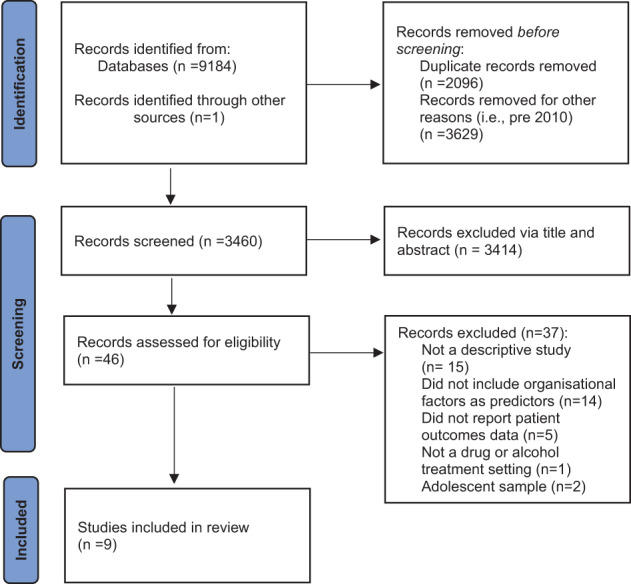
PRISMA flow chart.

After duplicates were removed, a total of 3459 citations were retrieved from the database search. Initial title and abstract review indicated 46 potentially relevant articles, of which 9 met the eligibility criteria and were included in the review (see Figure 1).

### 3.2 Study characteristics

Six studies were conducted in the United States, one of which included participants from the United States and Sweden [36], one study was conducted in the United Kingdom [37], one in South Africa [38] and one in Australia [39]. Eight studies were conducted in substance abuse treatment units or programs [37, 38, 40, 41, 42, 43, 44], while one study included AOD treatment centres but excluded centres focussed specifically on other drug use (i.e., those that did not include treatment for alcohol use) [36]. Four studies included outpatient treatment units only [37, 40, 41, 42] and five included both inpatient or residential and outpatient treatment programs [36, 38, 43, 44]. The number of centres included in the studies ranged from 6 to 363 and the number of participants ranged from 124 to 8599. Five studies were cross‐sectional [38, 40, 41, 42] and three used a prospective longitudinal design with data collected at either 3‐ [37], 6‐ [43] or 12‐month follow‐up [36, 44].

### 3.3 Methodological quality of included studies

Table 2 summarises the methodological quality of included studies. Two studies scored ‘Yes’ on all Joanna Briggs criteria [39, 41]. All studies scored ‘Yes’ across four out of eight criteria, including: ‘study subjects and setting described in detail’; ‘confounding factors identified’; ‘strategies to deal with confounding factors stated’; and ‘appropriate statistical analysis’. ‘Outcomes measured in a valid and reliable way’ was the least frequently met criterion, with only four studies scoring ‘Yes’ for this criterion [37, 39, 41, 42].

### 3.4 Organisational factors examined

Three studies examined the impact of cultural competency on treatment outcomes [40, 41, 42]. All three studies explored cultural competence in relation to African American and/or Mexican American people. Two of these studies assessed competency using the Cultural Competence Self‐Assessment Questionnaire [41, 42], while one developed a tool to examine organisational practices for cross‐cultural training, language congruence, diversity of staff, the availability of same‐race individual and group counselling, and the managers' culturally sensitive beliefs [40]. Guerrero and Andrews and Guerrero et al. also included a number of organisational control variables in their study (see Table 3) [40, 41]. For example, Guerrero et al. explored organisational readiness for change and directorial leaderships [41]. Guerrero et al. examined the impact of cultural competency alone, without including control variables [42].

**TABLE 3:** Data extraction for included studies.

Author, year; country	Setting and data collection method	Eligibility (for centres and participants)	Sample (total number of participants, age and gender)	Treatment seeking substance	Organisational predictors	Outcome measures and follow‐up time point(s)	Findings
Guerrero, 2011; USA	**Setting:** OSAT units. **Method:** Used Wave IV of the National Drug Abuse Treatment Service Survey—a longitudinal study examining the organisational structure and characteristics of OSAT units.	**Centres:** *Inclusion*: served a significant proportion of African American and Latino clients (demarcated at 20% and 15% respectively). **Participants:** N/A.	**Centres:** *N* = 363. **Participants:** N/A.	General substances.	Culturally competent practices (cross‐cultural training, language congruence, diversity of staff, and availability of same‐race individual and group counselling). Managers' culturally sensitive beliefs. *Control variables*: Organisational size (total clients served in past fiscal year); Location (urban or other); Staffing resources (ratio staff to clients); Service comprehensiveness (number of services offered by organisation); Ownership (private for‐profit, private non‐profit, or public); Affiliation (organisation is freestanding or part of a larger mental health or hospital setting).	**Outcome:** Retention in number of months (single survey item asking managers to estimate the average number of months clients stay in treatment). **F/U**: N/A.	The managers' beliefs scale was associated with an increase in average retention (*β* = 0.002, *p* < 0.01). The culturally competent practices measure was not associated with average retention. *Control variables*: Unit size was positively associated with average retention (*β* = 0.013, *p* < 0.05). Number of services provided was negatively associated with average retention (*β* = −0.004, *p* < 0.01)
Guerrero, 2016; USA	**Setting:** OSAT programs. **Method:** Client data was drawn from the Los Angeles County Participant Reporting System. Key‐informant data was also collected from clinical supervisors and cross‐validated with survey measures during follow‐up site visits.	**Centres:** *Inclusion*: publicly funded and non‐profit outpatient substance abuse treatment programs from communities with a population of 40% or more Black or Latino residents or both in Los Angeles County. *Exclusion*: programs involving inpatient or residential treatment, the criminal justice system or single practitioners. **Participants:** NR.	**Centres:** *N* = 97 **Participants:** *N* = 8599. Age: NR. Gender: NR.	General substances	Organisational readiness for change (Texas Christian University Organisational Readiness for Change) including motivation for change, resources and staff attributes. Directorial leadership (nine items assessing agency or program director leadership). Cultural competence (Cultural Competence Self‐Assessment Questionnaire). *Control variables*: State licensure. Accreditation by the Joint Commission.	**Outcomes**: Days in treatment (number of days between admission and discharge dates as noted by counsellors). **F/U:** N/A.	*Positively associated with treatment duration*: readiness for change measured by motivation for change (IRR 1.011, *p* < 0.05); The latent variable representing high‐program capacity (high leadership, readiness for change, and having a Medi‐Cal payment system) (IRR 1.295, *p* < 0.001); Programs with high cultural competence (IRR 1.157, *p* < 0.001); Licensed programs (IRR 1.148, *p* < 0.05). Staff attributes were negatively associated with treatment duration (IRR 0.979, *p* < 0.01). *Control variables*: Accreditation had no relationship with treatment duration.
Guerrero, 2017; USA	**Setting:** OSAT programs. **Method:** Analysis of a concatenated dataset from included treatment programs.	**Centres:** *Inclusion*: publicly funded and non‐profit outpatient substance abuse treatment programs from communities with a population of 40% or more Black or Latino residents or both in Los Angeles County. *Exclusion*: programs involving inpatient or residential treatment, the criminal justice system, or single practitioners. **Participants:** *Included*: Mexican‐American and non‐Latino White clients.	**Centres:** *N* = 153 **Participants:** *N* = 15,412. Age: NR Gender: NR.	General substances	Cultural competence (Cultural Competence Self‐Assessment Questionnaire).	**Outcomes:** Successful SUD treatment completion based on three indicators: (a) the client reported no alcohol or drug use during the 30 days prior to discharge; (b) the clinician reported client sobriety at discharge; and (c) the clinician coded treatment episode as successful based on the client meeting treatment goals for that episode. **F/U:** N/A.	No relationship between high implementation of cultural competence for Mexican Americans with successfully completing SUD treatment when compared with non‐Latino Whites and programs with low implementation of cultural competence (OR 1.185; 95% CI 0.834, 1.683; *p* = 0.344).
Myers, 2022; South Africa	**Setting:** Inpatient and residential substance use disorder services in the Western Cape Province. **Method:** Cross‐sectional study of clinical record and patient survey data.	**Centres:** *Inclusion*: substance use treatment centres included in the Service Quality Measures performance measurement system. *Exclusion*: NR. **Participants:** *Included*: patients attending the included centres.	**Centres:** *N* = 32. **Participants:** *N* = 1097. Age: 33.4 (SD ± 10.1). Gender: 73.7% male.	General substances.	Source of funding for treatment (state versus other)	**Outcomes:** Patient‐reported suboptimal outcomes of SUD treatment. **F/U:** N/A	State‐funded treatment was not associated with patient perceptions of suboptimal SUD treatment outcomes.
Ritter, 2021; Australia	**Setting:** AOD treatment services that receive government funding. **Method:** Cross‐sectional multi‐level analysis via manager survey and treatment centre records.	**Centres:** *Inclusion*: AOD sites with episodes of care recorded in the 2016/17 Australian Institute of Health and Wellbeing database. *Exclusion*: sites that had merged since identification via the database; specialised services for Aboriginal and Torres Strait Islander people; and assessment‐only services. **Participants:** *Included*: episodes of care within participating centres.	**Centres:** *N* = 178. **Participants:** *N* = 69,771 episodes of care. Age: NR. Gender: NR.	General substances.	Competitive tendering; number of funding sources; recurrent funding; episodes‐to‐staff ratio; turnover rates; government vs non‐government organisation for organisations covering each site.	**Outcomes:** Length of stay (continuous variable in days); and successful treatment completion (dichotomous variable where reason for treatment cessation is provide in database). **F/U:** N/A.	Having a lower episode‐to‐staff ratio (i.e., lower case‐loads) was associated with a longer length of stay (IRR 0.99, *p* = 0.047). Non‐government was associated with greater successful treatment completion (OR 0.34, *p* = 0.023). There were no other significant findings for organisational predictor variables for either outcome.
Schaefer, 2011; USA	**Setting:** Inpatient/residential and outpatient Veterans Affairs SUD treatment programs. **Method:** Discharge data drawn from the Veterans Affairs National Patient Care Database and self‐report measures were obtained from staff and clients.	**Centres:** *Inclusion*: inpatient/residential and intensive outpatient Department of Veterans Affairs SUD treatment programs. *Exclusion*: methadone maintenance programs. **Participants:** NR.	**Centres:** 28 (10 inpatient/residential and 18 outpatient) **Participants:** *N* = 865 Age = 47.22 (SD ± 7.79) Gender = 98% male.	General substances.	Continuity of Care Practices Survey, which had four subscales: Coordinate care; Connect to resources; Maintain contact; and Provider continuity.	**Outcome:** Engagement in continuing care: number of consecutive months after discharge that the patient had 2+ visits and no psychiatric inpatient admissions. Abstinence from alcohol and other drugs: addiction Severity Index‐Psychiatric. **F/U:** 6 months.	Patients receiving more continuity of care services engaged in continuing care longer (*β* = 0.39, *p* ≤ 0.001). Continuity of care service scores were not related to abstinence; however, each additional consecutive month of twice‐monthly SUD or psychiatric clinic visits was associated with an increased likelihood of abstinence (OR 1.2, *p* < 0.001).
Schulte, 2010; UK	**Setting:** Outpatient drug and/or alcohol treatment centres. **Method:** Client and staff assessments were conducted and retention was measured at 90 days.	**Centres:** *Inclusion*: urban and non‐urban outpatient drug and/or alcohol treatment programs in North West England. *Exclusion*: NR. **Participants:** *Inclusion*: clients attending participating centres with coexisting mental health problems and their responsible practitioners. *Exclusion*: NR.	**Centres:** 6. **Participants:** *Patients*: *N* = 124. Age: 39.0 (SD ± 9.8). Gender: 76% male. *Practitioners*: *N* = 46. Age: 40.2 (SD ± 7.0). Gender: 67% female.	General substances.	DD training received DD work experience DD competency Therapeutic optimism	**Outcome:** Retention rates of DD clients in outpatient addiction treatment. **F/U:** 90 days.	DD training and work experience did not meet inclusion for the final multi‐variate model (i.e., *p* > 0.25). DD competency was a significant predictor of retention (HR 0.657, *p* = 0.010). Therapeutic optimism was not significantly related to retention.
Shin, 2011; USA	**Setting:** Residential and non‐residential substance abuse treatment programs. **Method:** A secondary analysis of data collected during 1992–1995 for the National Treatment Improvement Evaluation Study, a prospective, cohort‐based study of USA substance abuse treatment programs and their clients.	**Centres:** *Inclusion*: treatment organisations serving vulnerable and underserved populations, including minorities, pregnant women, youth, public housing residents, welfare recipients and those involved in the criminal justice system. **Participants:** *Inclusion*: completed all intake, treatment discharge and follow‐up interviews. *Exclusion*: clients from correctional facilities; clients who reported no service need at intake.	**Centres:** 59. **Participants:** *N* = 3027. Age: 32.28 (SD ± 8.64). Gender: 63% male.	General substances	Access services (transportation and child care). Service‐needs ratio. *Control variables*: accreditation, treatment setting, ownership, on‐site service availability, and frequency of individual and group counselling.	**Outcome:** Treatment duration: length between first and last days of treatment. Post‐treatment drug use: number of days in the last 30 that respondent reporting using substances. **F/U:** treatment entry, exit and 12 months post‐treatment.	For non‐residential settings, service‐needs ratio predicted longer treatment duration and reduced post‐treatment substance use. In residential settings, service‐needs ratio is directly related to longer treatment duration.
Witbrodt, 2012; Sweden/USA	**Setting:** Inpatient and outpatient alcohol and drug treatment centres in Sweden and the USA. **Method:** A cross‐cultural analysis using a comparative contrast strategy and involving structured interviews with clients enrolled in treatment programs.	**Centres:** *Inclusion*: USA sample—public or private programs whose focus was not primarily drug abuse; had at least one intake per week; and were the first line treatment entry. *Exclusion*: USA sample—aftercare programs. **Participants:** *Inclusion*: new admissions presenting for services at study recruitment sites; ≥18 years old; ability to complete an in‐person structured interview in the native language. *Exclusion*: Swedish sample—Individuals recruited from methadone treatment or drug detoxification sites.	**Centres:** USA sample = 10; Swedish sample = approx. 20 (though patients from methadone or detoxification sites excluded so final inclusion unknown). **Participants:** *N* = 1498. Age: (Swedish) 18–34: 17%; 35–49: 39%; 50+: 44%. (US) 18–34: 30%; 35–49: 54%; 50+: 16%. Gender: (Swedish) 74% male; (US) 62% male.	Alcohol	Swedish sample: receiving treatment within: (i) the health‐care system; (ii) the social welfare system; or (iii) being assessed by the social welfare system but referred outside of it. USA sample: treatment in public versus private program.	**Outcome:** Questions from the Graduated Frequency Scale were used to create drinking typology outcome. This involved a yearly drink volume used to determine level of drinking (moderate, heavy and abstainer). **FU:** Baseline and 1 year.	Swedish sample: clients receiving treatment within the social welfare system were less likely to be moderate than heavy drinkers compared to the healthcare system (OR 0.27; [0.12, 0.63], *p* < 0.01). Those from the social welfare system who were referred to an outside treatment program were more likely to be abstainers than heavy drinkers than those in the healthcare system (OR = 6.09 [3.10, 12.0], *p* < 0.001). USA sample: clients treated in public treatment programs were less likely to be moderate than heavy drinkers (OR 0.56 [0.29, 0.89], *p* ≤ 0.05). The same directional trend emerged comparing abstainers to heavy drinkers (OR 0.58 [0.34, 1.00], *p* ≤ 0.05)

Abbreviations: CI, confidence interval; DD, dual diagnosis; F/U, follow up; HR, hazard ratio; IRR, incidence rate ratio; N/A, not applicable; NR, not reported; OR, odds ratio; OSAT: outpatient substance abuse treatment; SUD, substance use disorder.

One study examined continuity of care on treatment outcomes across four domains (coordinate care; connect to resources; maintain contact; and provider continuity) [43]. Another study examined service access variables (availability of transportation and child care) and the service to needs ratio of organisations (number of services clients said they received compared with the number they said they needed) [44]. This study included several organisational control variables in the analysis (e.g., accreditation and private ownership) (see Table 3). One study examined dual diagnosis training, work experience, competency and therapeutic optimism [37]. Three studies examined funding systems of the AOD settings, including state versus other funding [38], the funding systems in two countries (Sweden and United States) [36] and competitive tendering, number of funding contracts, recurrent funding and type of provider (government vs. non‐government) [39]. For the Swedish sample, factors examined included whether patients received treatment within the health‐care system; the social welfare system; or were being assessed by the social welfare system but referred outside of it. In the US sample, the study examined whether patients were treated in a public or private program. In addition to funding systems, one study also explored ratios of episodes to staff and staff turnover [39].

### 3.5 Outcome measures

Six studies examined treatment retention as an outcome [37, 39, 40, 41, 42, 44]. Guerrero and Andrews examined retention in number of months, which was measured via a single survey item asking managers to estimate the average number of months clients stay in treatment [40]. Guerrero et al., Shin et al. and Ritter et al. examined days in treatment via the number of days between admission and discharge dates [39, 41, 44]. Ritter et al. also examined successful treatment completion by examining whether treatment cessation was recorded [39]. Guerrero et al. examined successful treatment completion, for substance use disorder, based on three indicators: (i) the client reported no alcohol or drug use during the 30 days prior to discharge; (ii) the clinician reported client sobriety at discharge; and (iii) the clinician coded treatment episode as successful based on the client meeting treatment goals for that episode [42]. Schulte et al. defined dropout as failure to attend two or more sessions without referral to another service [37].

Three studies examined substance use as an outcome [36, 43, 44]. Schaefer et al. examined abstinence from AOD using the Addiction Severity Index‐Psychiatric [43]. Myers et al. used a patient reported outcome measure to examine patient perceptions of their treatment outcomes [38]. Along with treatment retention, Shin et al. examined post‐treatment drug use via the number of days in the past month that respondents reported using substances [44]. Witbrodt and Romelsjö used questions from the Graduated Frequency Scale to create a drinking typology outcome of ‘abstainer’, ‘moderate’ or ‘heavy’ drinking levels [36]. Schaefer et al. examined abstinence from AOD, as well as engagement in continuing care, measured by the number of consecutive months after discharge that the patient had two or more clinic visits with no SUD or psychiatric inpatient admissions [43].

### 3.6 Findings

Seven of the nine studies found significant associations between the organisational factors examined and treatment outcomes [36, 37, 39, 40, 41, 43, 44]. Guerrero and Andrews found the managers' belief scale was associated with an increase in average retention, however, culturally competent practices were not [40]. Guerrero et al. found readiness for change; high‐program capacity (high leadership, readiness for change and having Medicaid); high cultural competence; and licensed programs to be significantly associated with increased treatment duration [41]. Staff readiness for change was negatively associated with treatment duration and accreditation had no relationship with treatment duration [41]. Ritter et al. found a lower episode‐to‐staff ratio (i.e., lower case‐loads) was associated with a longer length of stay and non‐government organisation providers were associated with greater successful treatment completion [39]. Schaefer et al. found patients receiving a higher level of continuity of care services engaged in care for a longer duration [43]. Schulte et al. found practitioners' rating of their dual diagnosis treatment competency was a significant predictor of retention [37].

Shin et al. found that in non‐residential settings, service‐needs ratio predicted longer treatment duration and reduced post‐treatment substance use [44]. In residential settings, the service‐needs ratio was related to longer treatment duration but not post‐discharge substance use. Witbrodt and Romelsjö found in the US sample, clients treated in public treatment programs were less likely to be abstainers or moderate drinkers versus heavy drinkers when compared to private programs [36]. In the Swedish sample, when compared to being treated in the health‐care system, clients receiving treatment within the social welfare system were less likely to be moderate than heavy drinkers. Those from the social welfare system who were referred to an outside treatment program were more likely to be abstainers than heavy drinkers. Guerrero et al. and Myers et al. did not identify any significant associations in their respective studies [38, 42].

## 4 DISCUSSION

This systematic review is the first to comprehensively examine the association between organisational factors and AOD treatment outcomes. While only nine studies were identified, examining a range of organisational factors, seven of these found a statistically significant relationship between at least one organisational factor and client treatment outcomes. The findings suggests that organisational factors may impact AOD treatment outcomes. However, the wide variation in the organisational factors examined, study settings and measures used across the nine studies make it difficult to draw conclusions on the importance of specific factors.

Factors such as unit size, provided services, continuity of care, staff ratios and funding model were reported to be associated with AOD outcomes. This aligns with research on organisational factors in other health‐care settings, such as primary and acute care settings [26, 45]. However, a range of organisational factors which have been identified as important in other settings were not explored in any of the identified studies, including urban location and teaching status. Further, higher case‐loads and higher staff to patient ratios were only explored in one recent study [39]. Similar conclusions were drawn in a recent review by van de Ven et al., which only identified two studies examining staff turnover, both focussed on adolescent samples, and four studies examining staff‐to‐client ratios, all of which were conducted prior to 2007 [31]. While the current review included an increased number of organisational factors, both the current and previous review highlight a need for future research to consider methodologies that examine a core set of organisation factors used consistently across studies.

Across the nine included studies, a variety of AOD treatment outcome measures were used to examine their association with organisational factors. These included treatment duration, completion or continuation; AOD use; and patient perceptions of treatment outcomes. While retention and/or completion are important outcomes of AOD treatment, these represent proxy measures for treatment effectiveness rather than explicit AOD outcomes, such as abstinence or a reduction in use. In addition, only three studies used the same measurement to assess the AOD treatment outcomes (i.e., treatment duration [39, 41, 44]), and only one study examining AOD use used a clearly validated measure [43]. The importance of measuring study outcomes using valid and reliable measures is well‐established [46], however, issues surrounding the use of standardised measures for AOD treatment outcomes have been raised as problematic [47]. The use of the same measure across studies enables comparison and synthesis. Although potentially more difficult and costly to capture, future studies should endeavour to include AOD use as the primary outcome and consider measures that can be used consistently across studies.

Five of the included studies involved both inpatient and outpatient treatment settings, while the remaining included outpatient only. Eight studies involved general substance use, with one study focussed on alcohol use only [36]. Most of the included studies were conducted in the United States, with one including a Swedish sample, and three others conducted in South Africa, the United Kingdom and Australia. Given the organisational and environmental structure of treatment facilities are likely to vary widely between countries, it is unlikely these findings can be generalised across countries. This review highlights a need to increase research studies exploring organisational factors in countries other than the United States, as well as a potential to increase the research conducted within inpatient treatment settings.

### 4.1 Limitations

There was considerable effort to ensure the search strategy was comprehensive, however, the review does not include research conducted prior to 2010. This date was chosen to ensure the findings were relevant for current practice and organisational structures, however, studies examining organisational factors prior to this date will have been excluded due to this. The search strategy was based on the Theoretical Domains Framework to ensure a broad and inclusive search was applied; nevertheless, it is possible that some relevant studies may have been missed. As the included studies were descriptive rather than experimental, the associations found cannot be used to attribute causality. Exclusion of non‐English studies was due to pragmatic issues in study translation and may have resulted in relevant non‐English studies being excluded.

### 4.2 Future research

This review highlights that examining the impact of organisational factors is an important avenue for future research. Further rigorous descriptive studies are needed to understand the relationship between organisational factors and AOD treatment outcomes. Such information can be used to inform future policies, practice and intervention research. Future research should endeavour to include consistent and validated measures of organisational factors and AOD treatment outcomes.

## 5 CONCLUSIONS

As with other health‐care settings, this systematic review found that organisational factors may have an impact on outcomes for people seeking treatment for AOD use. The heterogeneity in organisational factors, treatment outcomes and study setting prohibits firm conclusion about which organisational factors are most important for outcomes.

## AUTHOR CONTRIBUTIONS

Each author certifies that their contribution to this work meets the standards of the International Committee of Medical Journal Editors.

## CONFLICT OF INTEREST STATEMENT

None to declare.

## Supporting information


**Data S1:** Supporting Information
