# Supplementary material for: The impact of organisational factors on treatment outcomes for those seeking alcohol or other drug treatment: A systematic review
Source: Drug Alcohol Rev. 2023 Apr 2;42(5):1220–34. doi: 10.1111/dar.13653 (PMC10947488; doi:10.1111/dar.13653)
Supplement: Supplementary file 1 — Data S1: Supporting Information [file DAR-42-1220-s001.docx]

**Appendix S1**

Database: Ovid MEDLINE(R) and Epub Ahead of Print, In-Process, In-Data-Review & Other Non-Indexed Citations and Daily <1946 to March 08, 2022>

Search Strategy:

--------------------------------------------------------------------------------

1 Alcoholism/rh, th (16464)

2 Alcohol drinking/pc, th (5362)

3 Binge drinking/pc, rh, th (279)

4 Alcohol-related disorders/pc, rh, th or Substance-related disorders/rh, th, pc (31227)

5 Drinking behavior/ (6860)

6 (drink* adj2 (behavio?r* or problem* or hazardous or heavy or harmful or at risk or high risk or binge)).ti,ab,kw,kf. (26165)

7 (alcohol* adj3 (treat* or therap* or rehabilitat* or reduc* or depend* or prevent* or control* or program*)).ti,ab,kw,kf. (51079)

8 1 or 2 or 3 or 4 or 5 or 6 or 7 (111915)

9 "Organization and administration"/ or decision making, organizational/ or organizational culture/ or organizational innovation/ or organizational objectives/ or organizational policy/ or models, organizational/ or total quality management/ (119400)

10 "Delivery of Health care"/ or "Delivery of Health Care, Integrated"/ (118852)

11 Quality of health care/ (76387)

12 Leadership/ (45102)

13 health facility administrators/ or hospital administrators/ or chief executive officers, hospital/ or nurse administrators/ or physician executives/ (29035)

14 "Attitude of Health Personnel"/ (129056)

15 exp Patient care team/ (71871)

16 exp Interprofessional relations/ (71860)

17 Cooperative Behavior/ (45490)

18 Professional-patient relations/ (28297)

19 exp Professional competence/ (125635)

20 Practice patterns, nurses'/ or practice patterns, physicians'/ or professional practice gaps/ (68502)

21 Education/ or exp education, professional/ or exp inservice training/ (360107)

22 exp health personnel/ed (63649)

23 health service administration/ or hospital administration/ or health facility administration/ or exp health facility environment/ (39147)

24 exp workforce/ (78882)

25 Patient-centered care/ (21855)

26 Professional practice/ (17010)

27 exp financial management/ or exp financial support/ or financing, organized/ (138013)

28 Financing, government/ (21282)

29 cost-benefit analysis/ or health care costs/ or health expenditures/ (140535)

30 health financing/ or hospitals/ec (1144)

31 Health workforce/ (14062)

32 Health plan implementation/ (6623)

33 "Diffusion of Innovation"/ or Implementation Science/ (19262)

34 Cultural competence/ (6221)

35 Guideline adherence/ (34627)

36 "Referral and Consultation"/ (72575)

37 Physician Incentive Plans/ (2283)

38 Reimbursement, Incentive/ (4663)

39 incentivi?ed care.ti,ab,kw,kf. (5)

40 og.fs. (503907)

41 ((organi?ation* or service or management or program*) adj3 (determinant* or practice* or variable* or characteristic* or factor* or structure* or mission* or philosoph* or predictor* or prediction* or barrier* or enabler* or facilitator* or policy or policies or climate or delivery or quality)).ti,ab,kw,kf. (188642)

42 or/9-41 (1760280)

43 8 and 42 (13314)

44 treatment outcome/ or treatment failure/ (1120913)

45 exp "outcome and process assessment (health care)"/ (1299692)

46 (outcome* adj2 (treatment* or patient* or care or drinking)).ti,ab,kw,kf. (326872)

47 (abstain* or abstinen*).ti,ab,kw,kf. (33219)

48 (alcohol* adj2 (reduc* or cessation or ceas* or re-laps* or relaps* or recurrence* or re-currence*)).ti,ab,kw,kf. (9551)

49 (treatment adj effect*).ti,ab,kw,kf. (44941)

50 (dependence adj severity).ti,ab,kw,kf. (302)

51 Program evaluation/ (66456)

52 44 or 45 or 46 or 47 or 48 or 49 or 50 or 51 (1627245)

53 43 and 52 (3571)

54 limit 53 to english language (3418)

55 limit 54 to (case reports or comment or editorial or letter or news) (98)

56 54 not 55 (3320)

Database: Embase <1947 to present>

Search Strategy:

--------------------------------------------------------------------------------

1 *alcoholism/ (84604)

2 *drinking behavior/ (17577)

3 *binge drinking/ (1911)

4 *alcohol consumption/ (32899)

5 *drug dependence/ (34598)

6 *substance abuse/ (19078)

7 (drink* adj2 (behavio?r or problem* or hazardous or heavy or harmful or risk or binge)).ti,ab,kw. (35210)

8 (alcohol* adj3 (treat* or therap* or rehabilitat* or reduc* or depend* or prevent* or control* or program*)).ti,ab,kw. (71658)

9 1 or 2 or 3 or 4 or 5 or 6 or 7 or 8 (220527)

10 "organization and management"/ (429326)

11 organization/ (159121)

12 organizational culture/ (2615)

13 organization/ or organizational efficiency/ or organizational structure/ (162914)

14 organizational policy/ (1768)

15 health care management/ (31198)

16 total quality management/ (77858)

17 health care delivery/ (194866)

18 health care quality/ (256902)

19 leadership/ (80524)

20 health care facility/ (81210)

21 exp hospital administrator/ (9935)

22 health personnel attitude/ (87971)

23 patient care/ (328451)

24 cooperation/ (45503)

25 public relations/ (63440)

26 exp competence/ (132668)

27 clinical practice/ (324109)

28 practice gap/ (930)

29 in service training/ (16697)

30 continuing education/ (32853)

31 professional development/ (11302)

32 health care personnel/ (198369)

33 professional practice/ (58459)

34 financial management/ (121323)

35 health care financing/ (13653)

36 "cost benefit analysis"/ (89893)

37 "health care cost"/ or health care financing/ or "hospital cost"/ (236396)

38 cultural competence/ (7307)

39 protocol compliance/ (17629)

40 patient referral/ (136837)

41 reimbursement/ (61736)

42 incentivi?ed care.ti,ab,kw. (15)

43 ((organi?ation* or service or management or program*) adj3 (determinant* or practice* or variable* or characteristic* or factor* or structure* or mission* or philosoph* or predictor* or prediction* or barrier* or enabler* or facilitator* or policy or policies or climate or delivery or quality)).ti,ab,kw. (243657)

44 or/10-43 (2538279)

45 9 and 44 (14639)

46 treatment outcome/ or treatment failure/ (1037380)

47 outcome assessment/ (649335)

48 (outcome* adj2 (treatment* or patient* or care or drinking)).ti,ab,kw. (514678)

49 (abstain* or abstinen*).ti,ab,kw. (44450)

50 (alcohol* adj2 (reduc* or cessation or ceas* or re-laps* or relaps* or recurrence* or re-currence*)).ti,ab,kw. (13767)

51 (treatment adj effect*).ti,ab,kw. (67050)

52 (dependence adj severity).ti,ab,kw. (425)

53 harm reduction/ (7671)

54 exp program evaluation/ or evaluation study/ (81381)

55 46 or 47 or 48 or 49 or 50 or 51 or 52 or 53 or 54 (2152447)

56 45 and 55 (3460)

57 limit 56 to english language (3278)

58 limit 57 to (books or chapter or conference abstract or conference paper or "conference review" or editorial or letter or note) (653)

59 57 not 58 (2625)

Database: APA PsycInfo <1806 to February Week 4 2022>

Search Strategy:

--------------------------------------------------------------------------------

1 exp alcohol abuse/ or alcohol intoxication/ (52795)

2 exp "substance use treatment"/ (30543)

3 exp "substance use disorder"/ (138105)

4 (drink* adj2 (behavio?r or problem* or hazardous or heavy or harmful or at risk or high risk or binge)).ti,ab,id. (21771)

5 (alcohol* adj3 (treat* or therap* or rehabilitat* or reduc* or depend* or prevent* or control* or program*)).ti,ab,id. (37663)

6 1 or 2 or 3 or 4 or 5 (162921)

7 exp organizational characteristics/ or exp organizational behavior/ or organizational commitment/ or organizational learning/ or organizational objectives/ (86253)

8 organizational climate/ or organizational politics/ or exp organizational structure/ (19072)

9 organizations/ (27860)

10 exp health care delivery/ (106536)

11 leadership/ or leadership qualities/ or leadership style/ (47003)

12 management personnel/ (23800)

13 health personnel attitudes/ (21435)

14 management/ or management decision making/ or work teams/ (23722)

15 teams/ (12991)

16 collaboration/ (13036)

17 exp competence/ (28625)

18 professional development/ or continuing education/ or inservice training/ (23414)

19 health personnel/ (18855)

20 finance/ or funding/ or "costs and cost analysis"/ or resource allocation/ (30436)

21 cultural sensitivity/ or cross cultural communication/ or cross cultural differences/ or cross cultural treatment/ (63810)

22 professional referral/ (3708)

23 incentives/ (5230)

24 ((organi?ation* or service or management or program*) adj3 (determinant* or practice* or variable* or characteristic* or factor* or structure* or mission* or philosoph* or predictor* or prediction* or barrier* or enabler* or facilitator* or policy or policies or climate or delivery or quality)).ti,ab,id. (107622)

25 7 or 8 or 9 or 10 or 11 or 12 or 13 or 14 or 15 or 16 or 17 or 18 or 19 or 20 or 21 or 22 or 23 or 24 (514404)

26 exp treatment outcomes/ (131076)

27 evaluation/ or exp treatment effectiveness evaluation/ (46354)

28 program evaluation/ (13296)

29 (outcome* adj2 (treatment* or patient* or care or drinking)).ti,ab,id. (51226)

30 (abstain* or abstinen*).ti,ab,id. (23862)

31 (alcohol* adj2 (reduc* or cessation or ceas* or re-laps* or relaps* or recurrence* or re-currence*)).ti,ab,id. (6138)

32 (treatment adj effect*).ti,ab,id. (18552)

33 (dependence adj severity).ti,ab,id. (324)

34 26 or 27 or 28 or 29 or 30 or 31 or 32 or 33 (244704)

35 6 and 25 and 34 (3500)

36 limit 35 to english language (3365)

37 limit 36 to (chapter or "column/opinion" or "comment/reply" or dissertation or editorial or letter) (633)

38 36 not 37 (2732)

Search Name: TDF

Date Run: 10/03/2022 12:24:27

Comment:

ID Search Hits

#1 MeSH descriptor: [Alcoholism] explode all trees and with qualifier(s): [rehabilitation - RH, therapy - TH] 1427

#2 MeSH descriptor: [Alcohol Drinking] this term only and with qualifier(s): [prevention & control - PC, therapy - TH] 1062

#3 MeSH descriptor: [Binge Drinking] this term only and with qualifier(s): [prevention & control - PC, rehabilitation - RH, therapy - TH] 103

#4 MeSH descriptor: [Alcohol-Related Disorders] this term only and with qualifier(s): [prevention & control - PC, rehabilitation - RH, therapy - TH] 266

#5 MeSH descriptor: [Substance-Related Disorders] this term only and with qualifier(s): [prevention & control - PC, rehabilitation - RH, therapy - TH] 1967

#6 MeSH descriptor: [Drinking Behavior] this term only 146

#7 (drink* near/2 (behavior* or behaviour* or problem* or hazardous or heavy or harmful or "at risk" or "high risk" or binge)):ti,ab 4395

#8 (alcohol* near/5 (treat* or therap* or rehabilitat* or reduc* or depend* or prevent* or control* or program*)):ti,ab 11251

#9 #1 or #2 or #3 or #4 or #5 or #6 or #7 or #8 14913

#10 MeSH descriptor: [Organization and Administration] this term only 42

#11 MeSH descriptor: [Decision Making, Organizational] this term only 42

#12 MeSH descriptor: [Organizational Culture] this term only 109

#13 MeSH descriptor: [Organizational Innovation] this term only 113

#14 MeSH descriptor: [Organizational Objectives] this term only 49

#15 MeSH descriptor: [Organizational Policy] this term only 97

#16 MeSH descriptor: [Models, Organizational] this term only 180

#17 MeSH descriptor: [Total Quality Management] this term only 139

#18 MeSH descriptor: [Delivery of Health Care] this term only 881

#19 MeSH descriptor: [Delivery of Health Care, Integrated] this term only 424

#20 MeSH descriptor: [Quality of Health Care] this term only 920

#21 MeSH descriptor: [Leadership] this term only 229

#22 MeSH descriptor: [Health Facility Administrators] this term only 8

#23 Hospital administrators 261

#24 MeSH descriptor: [Hospital Administrators] this term only 10

#25 MeSH descriptor: [Chief Executive Officers, Hospital] this term only 0

#26 MeSH descriptor: [Nurse Administrators] this term only 28

#27 MeSH descriptor: [Physician Executives] this term only 8

#28 MeSH descriptor: [Attitude of Health Personnel] this term only 2013

#29 MeSH descriptor: [Patient Care Team] explode all trees 1791

#30 MeSH descriptor: [Interprofessional Relations] explode all trees 596

#31 MeSH descriptor: [Cooperative Behavior] this term only 968

#32 MeSH descriptor: [Professional-Patient Relations] this term only 796

#33 MeSH descriptor: [Professional Competence] explode all trees 3886

#34 MeSH descriptor: [Practice Patterns, Nurses'] this term only 163

#35 MeSH descriptor: [Practice Patterns, Physicians'] this term only 1300

#36 MeSH descriptor: [Professional Practice Gaps] this term only 3

#37 MeSH descriptor: [Education] this term only 605

#38 MeSH descriptor: [Education, Professional] explode all trees 5333

#39 MeSH descriptor: [Inservice Training] explode all trees 831

#40 MeSH descriptor: [Health Personnel] explode all trees and with qualifier(s): [education - ED] 1955

#41 MeSH descriptor: [Health Services Administration] this term only 10

#42 MeSH descriptor: [Hospital Administration] this term only 26

#43 MeSH descriptor: [Health Facility Administration] this term only 1

#44 MeSH descriptor: [Health Facility Environment] explode all trees 128

#45 MeSH descriptor: [Workforce] explode all trees 425

#46 MeSH descriptor: [Patient-Centered Care] this term only 681

#47 MeSH descriptor: [Professional Practice] this term only 113

#48 MeSH descriptor: [Financial Management] explode all trees 267

#49 MeSH descriptor: [Financial Support] explode all trees 135

#50 MeSH descriptor: [Financing, Organized] this term only 20

#51 MeSH descriptor: [Financing, Government] this term only 49

#52 MeSH descriptor: [Cost-Benefit Analysis] this term only 7600

#53 MeSH descriptor: [Health Care Costs] this term only 2372

#54 MeSH descriptor: [Health Expenditures] this term only 247

#55 MeSH descriptor: [Healthcare Financing] this term only 8

#56 MeSH descriptor: [Health Workforce] this term only 23

#57 MeSH descriptor: [Health Plan Implementation] this term only 190

#58 MeSH descriptor: [Diffusion of Innovation] this term only 136

#59 MeSH descriptor: [Implementation Science] this term only 53

#60 MeSH descriptor: [Cultural Competency] this term only 187

#61 MeSH descriptor: [Guideline Adherence] this term only 1124

#62 MeSH descriptor: [Referral and Consultation] this term only 2029

#63 MeSH descriptor: [Physician Incentive Plans] this term only 15

#64 MeSH descriptor: [Reimbursement, Incentive] this term only 140

#65 ("incentivized care" or "incentivised care"):ti,ab 2

#66 ((organi?ation* or service or management or program*) near/3 (determinant* or practice* or variable* or characteristic* or factor* or structure* or mission* or philosoph* or predictor* or prediction* or barrier* or enabler* or facilitator* or policy or policies or climate or delivery or quality)) 22101

#67 [41-#66] 47340

#68 #9 and #67 1031

#69 MeSH descriptor: [Treatment Outcome] this term only 143126

#70 MeSH descriptor: [Treatment Failure] this term only 3417

#71 MeSH descriptor: [Outcome and Process Assessment, Health Care] explode all trees 161061

#72 (outcome* near/2 (treatment* or patient* or care or drinking)):ti,ab 68393

#73 (abstain* or abstinen*):ti,ab 11676

#74 (alcohol* near/2 (reduc* or cessation or ceas* or re-laps* or relaps* or recurrence* or re-currence*)):ti,ab 2945

#75 #69 or #70 or #71 or #72 or #73 or #74 227073

#76 #68 and #75 in Cochrane Reviews, Cochrane Protocols, Trials 560
